# Older Adults’ Identity Development and Mental Health After the COVID-19 Pandemic: A 4-Year Longitudinal Study

**DOI:** 10.1093/geroni/igaf122.1762

**Published:** 2025-12-31

**Authors:** Lauren Mitchell, Jessica Finlay

**Affiliations:** Emmanuel College, Boston, Massachusetts, United States; University of Colorado Boulder, Boulder, Colorado, United States

## Abstract

The COVID-19 pandemic introduced substantial challenges to older adults’ identities and self-concepts as they navigated social isolation, health risks, bereavement, and other disruptions. This mixed-methods study examined how older adults perceived their identities being affected by the pandemic, and how such identity shifts related to trajectories of well-being. Older adults (N = 2248; mean age=68) completed 12 monthly surveys in Year 1 (April, 2020-April, 2021) and annual surveys in Years 2-4 (2022-2024) of the COVID Coping Study (Kobayashi et al., 2021). Identity dynamics were assessed with open-ended questions addressing how the pandemic affected participants’ self-concepts, sense of purpose, and expectations for the future. Life satisfaction, loneliness, depressive symptoms, anxiety, and self-rated health were assessed with quantitative measures. Thematic analysis was used to identify common patterns among the open-ended identity responses, and latent growth curve modeling was used to test the association between identity themes and well-being outcomes. Initial findings suggest most participants emphasized positive identity adjustments, such as changing their priorities in life, or realizing their strength in the face of adversity. A small subset (6%) indicated identity disruption, or a loss of identity coherence and self-continuity, due to the pandemic. Identity disruption was associated with lower trajectories of mental health on average, and increasing levels of loneliness across the pandemic period. Findings suggest that, although most older adults have coped effectively with the identity-related challenges of the pandemic, some may continue to experience negative consequences that can hinder their social engagement and harm their mental health.

